# Uteroplacental insufficiency decreases forkhead box protein O1A (foxO1a) hepatic levels in perinatal and postnatal IUGR rats

**DOI:** 10.1186/1687-9856-2013-S1-P208

**Published:** 2013-10-03

**Authors:** Z Shen, CC Zou, SQ Shang, LZ Du

**Affiliations:** 1 Children’s Hospital, Zhejiang University School of Medicine, Hangzhou, Zhejiang, China; 2 Zhejiang Key Laboratory for Diagnosis and Therapy of Neonatal Diseases, Hangzhou, Zhejiang, China; 3 Key Laboratory of Reproductive Genetics(Zhejiang University), Ministry of Education, Hangzhou, Zhejiang, China

**Keywords:** IUGR, FoxO1a, Dietary restriction

## Objective

IUGR re-programs hepatic gene expression, leading to alterations in perinatal mRNA levels that persist postnatally. Forkhead bos protein O1a (FoxO1a) is a nclear transcription factor, which is an important regulator of the in vivo metabolism of energetic substance, and play an important role in stabilizing the hepatocyte glucose metabolism and maintaining blood glucose levels in the body. Recent study investigated FoxO1a expression can stimulate key enzymes of gluconeogenesis pathway phosphoenolpyruvate carboxylase kinases (PEPCK), peroxisome proliferator-activated receptor γ auxiliary activating factor-1α (PGC-1a) and glucose - 6 - phosphatase (G-6-Pase) gene expression. The effect of IUGR upon hepatic FoxO1a expression is unknown. So in this study we investigate the persistent changes in hepatic FoxO1a expression caused by IUGR, which could initiate and maintain the obesity, insulin resistance and type 2 diabetes, and explore whether the changes in FoxO1a expression are gender specific.

## Methods

Dietary restriction was used to induce uteroplacental insufficiency and subsequent IUGR. Control(CON) animals came from dams who received anesthesia. Pups were harvested at d0, d21 and d56 of postnatal life, and liver were harvested. Real-time RT-PCR and Western blotting were used to measure hepatic FoxO1a expression.

## Results

For female and male pups, IUGR significantly decreased FoxO1a hepatic mRNA and protein levels of CON values(*P*<0.01) at *day 0*, *day21* and at *day 56*.

## Conclusions

We conclude that IUGR decreases hepatic perinatal and postnatal FoxO1a expression in female and male rats. FoxO1a functions as a nclear transcription factor. Important future studies involve determining which genes are affected by this alteration response to IUGR.

